# Vitamin D Status and Its Association with Parathyroid Hormone Concentration in Brazilians

**DOI:** 10.1155/2017/9056470

**Published:** 2017-02-07

**Authors:** Juliana Sálvio Martins, Magda de Oliveira Palhares, Octávio Cury Mayrink Teixeira, Mariana Gontijo Ramos

**Affiliations:** College of Human, Social and Health Sciences, Universidade Fumec, Belo Horizonte, MG, Brazil

## Abstract

Vitamins are organic compounds that play a vital role in the control of metabolic processes. The D complex is considered a nutrient with a hormonal action and has an important participation in the constant maintenance of serum and extracellular calcium levels. The present study aims to analyze the results of 105.588 vitamin D (25(OH)D) measurements obtained from a database from a clinical analysis laboratory in Brazil, between the years of 2011 and 2013. The values of 25(OH)D were correlated with age, gender, and values of PTH. The results show a high prevalence of values of 25(OH)D considered inadequate, characterizing 76% of the studied population. It was observed that 26,5% of the individuals had deficiency and 49,5% had insufficiency of vitamin D. It was also shown that there was a negative correlation between 25(OH)D and PTH levels. In conclusion, this study is in accordance with others that show a high prevalence of vitamin D deficiency in different populations and alerts us for the importance of these measurements and analysis in clinical practice and as a base for diagnosis and treatment of hypovitaminosis.

## 1. Introduction

Vitamin D plays a key role in serum calcium homeostasis, acting as a hormone in autocrine and paracrine manners [1]. The main function of 1,25(OH)2D3, which is the active form of vitamin D, is to increase calcium absorption from the intestine, through interaction with vitamin D receptor (VDR), expressed in the distal as well as in the proximal intestine [2].

The major source of vitamin D is the skin synthesis, contributing to more than 90% of vitamin D serum concentration [1]. While absorbing solar energies (ultraviolet B), 7-dehydrocholesterol (provitamin D3) converts to vitamin D_3_ (cholecalciferol). Vitamin D can also be taken by diet, from fortified dairy products and fish oils [3, 4]. Vitamin D3 enters the circulation and is transported to the liver, where it is converted to 25-hydroxyvitamin D (25(OH)D), a form thought to be biologically inactive, but which is widely regarded as the best indicator of vitamin D status. In the kidney, 25(OH)D is metabolized to 1,25[OH]2D (calcitriol), which is the biologically active form of vitamin D [5].

Studies over the last decade have shown that the effects of vitamin D are not limited to the maintenance of calcium homeostasis. 1,25[OH]2D regulates multiple cellular processes, with effects on immune system, cardiovascular function, and interplay with other hormones [4].

Vitamin D deficiency can be caused by nutritional factors, decreased solar exposure, malabsorption resulting from intestinal inflammation, celiac disease or gastric surgery, prolonged use of anticonvulsants and corticoids, and others [6]. Low 25(OH)D status leads to reduced efficiency in intestinal calcium absorption, and the body reacts by increasing the secretion of parathyroid hormone (PTH) [7]. Especially in elderly people, increased serum PTH concentration can cause bone turnover and bone loss, defects in mineralization, and increased risk of fractures [8]. Furthermore, vitamin D supplementation with calcidiol, in addition to improving serum 25OHD, also significantly lowers PTH levels, reducing secondary hyperparathyroidism [9]. According to the existing literature, vitamin D deficiency not only affects bone metabolism and quality, but also seems to be related to autoimmune diseases, neurological diseases, and cancer [10]. Studies performed in different populations have shown that vitamin D deficiency is not restricted to northern countries where sunlight exposure is restricted, but could also be common in subtropical countries [11].

There is still no agreement on which levels of vitamin D should be considered normal or abnormal, and there are also differences in the methodologies used by laboratories to measure circulating vitamin D levels. However, there is an agreement that plasma levels of 25(OHO)D are the best indicator for vitamin D evaluation [12]. Although there is a variation of the ideal values to establish adequate levels of vitamin D, many experts agree that levels of 25(OH)D below 20 ng/mL are classified as deficient, levels between 20 and 29 ng/mL as insufficiency, and those between 30 and 100 ng/mL as sufficiency [13, 14]. The Brazilian Society of Endocrinology and Metabolism (SBEM) recommends that levels of 25(OH)D above 30 ng/mL are considered adequate and should be the goal for high-risk populations [13]. Low concentrations of serum 25(OH)D are suggested to be associated with high levels of parathyroid hormone (PTH) and a higher risk of mortality [15].

The present study aims to analyze the results of vitamin D laboratory test results obtained from a databank in southeast of Brazil and correlate the values with individual's age, gender, and PTH levels.

## 2. Materials and Methods

The present study is a descriptive cross-sectional study of data obtained through a databank from a clinical laboratory in Belo Horizonte, MG, Brazil. A total of 105588 25(OH)D tests results were collected between 2011 and 2013. Tests results from PTH measurements (13668) were also collected and analyzed. Samples were collected with a minimum eight hours fasting and vitamin D levels were obtained by quantitative determination of 25(OH)D using automated competitive chemiluminescence immunoassay method (Diasorin Liaison). The reference values used in the present study were those determined by the clinical laboratory as follows: <20 ng/mL (deficiency), 20–29 ng/mL (insufficiency), 30–100 ng/mL (sufficiency), and >100 ng/mL (increased). Parathyroid hormone levels were measured by direct chemiluminescence immunoassay method (ADVIA Centaur) with reference values range from 9 to 72 pg/mL.

Data were expressed as mean, standard deviation, median, and percentiles. Statistical analysis was performed using Mann–Whitney and Spearman nonparametric test. The significance level was set at 5% (*p* < 0.05).

Access to the clinical laboratory databank was previously authorized and the collected data were confidential. No personal information was obtained. The study was submitted and approved by the ethics committee from Universidade Fumec, protocol number 683.583.

## 3. Results

A total of 105588 laboratory test results were analyzed for 25(OH)D levels. It was possible to observe that the amount of serum 25(OH)D tests performed by the clinical analysis laboratory increased during the period of this study. In the first year, 16019 tests were performed, compared to 55571 in the last year of the study (data not shown).

The results from the analysis of all 25(OH)D tests results during the period of the study showed a mean value of 25.3 ± 9.3 ng/mL and median of 24.5 ng/mL. Data analysis also detected a value of 19.6 ng/mL for percentile 25 and of 29.7 ng/mL for percentile 75. The results were similar for each year analysis (Table 1).

**Figure 1:**
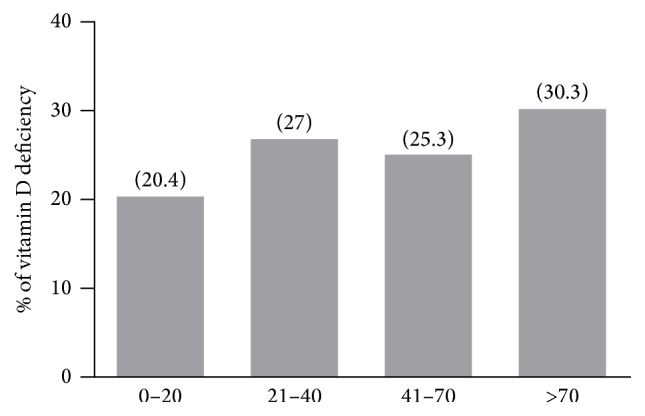
Percentage of vitamin D deficiency according to age group. Values lower than 20 ng/mL were considered as deficiency. Numbers in parenthesis show the exact percentage for each age group. A significant correlation was found between age and vitamin D deficiency (*p* < 0,001).

Statistical analysis of 25(OH)D values according to individuals age demonstrated that a negative correlation could be found between age and 25(OH)D values. The higher median values (26.1 ± 8.3 ng/mL) were observed for individuals between 0 and 20 years old, while those 70 years or older presented the lowest median values (4.7 ± 10.3 ng/mL) of vitamin D (data not shown). It was possible to note that 20.4% of individuals between 0 and 20 years old had 25(OH)D values lower than 20 ng/mL, while 30.3% of older than 70 individuals had values lower than 20 ng/mL, considered as vitamin D deficiency (Figure 1). The data suggest that vitamin D deficiency tends to increase with ageing.

Analysis of 25(OH)D measurements showed that 28065 (26.5%) of the total of tests results obtained were considered as deficient (less than 20 ng/mL), 52304 (49.5%) as insufficient (between 20 and 29 ng/mL), 25099 (23.8%) as sufficient (between 30 and 100 ng/mL), and 120 (0.11%) as increased (Table 2).

From the total 25(OH)D test results obtained, 13668 individuals also had results from PTH measurements. We could find a significative and negative correlation between levels of 25(OH)D and PTH (*p* < 0.0001). These data suggest that individuals with vitamin D deficiency show high PTH values (31.2 pg/mL), while those with vitamin D values above 100 ng/mL show lower levels of PTH (23.4 pg/mL) (Table 3). No significant differences were observed regarding the season when vitamin D was measured or the gender of the individuals.

## 4. Discussion

Vitamin D deficiency or insufficiency is currently an important nutritional and clinical concern all over the world, but there is still controversy about the optimal levels of serum 25(OH)D for maintaining bone and muscle health. According to the Endocrine Society directions, levels of 25(OH)D < 29 ng/mL are considered inadequate [16].

Over the last decade, there has been an increased interest in the role of vitamin D in health and diseases by the scientific community and by health professionals in general. This seems to be due to a deeper understanding of the importance of vitamin D not only in bone metabolism, but also in other cell signaling processes and body functions such as immune, nervous, and cardiovascular systems [17]. Insufficient levels of vitamin D are being correlated to an increased risk of cardiovascular, autoimmune diseases, diabetes, respiratory diseases, obesity, and cancer [17–19].

One of the consequences of the increased interest in hypovitaminosis D is the large number of requests for laboratory evaluation of circulating vitamin D levels. In the present study, it was possible to show an increase of more than 50% in the number of laboratory exams for vitamin D performed during the period of the study, from 2011 to 2013. Similar results were observed in an Australian population, with an increased number of laboratory test performed between 2001 and 2010. These authors describe a 730% increase in laboratory tests for vitamin D in 10 years [20]. Other clinical laboratories also detected more than 100% increase in the amount of vitamin D tests performed, as these tests are being requested to many individuals such as people with advanced age, pregnant women, obese people, patients with malabsorption syndromes, renal and liver problems, hyperparathyroidism, lymphomas, and others [13, 21].

In the present study a high prevalence of results showing inadequate levels of 25(OH)D was observed for around 76% of the studied population. Approximately 26.5% of the obtained values were classified as deficient and 49.5% as insufficient. The data analysis showed that 25% of the population seems to have vitamin D levels lower than 19.6 ng/mL and 75% lower than 29.7 ng/mL. These data are in accordance with the reference values found in the literature and to those we used in our study, further straightening the increased vitamin D deficiency in Brazilian individuals. Other studies performed in different countries also reinforce the crescent vitamin D deficiency, in both healthy and unhealthy people. Vierucci et al. showed insufficient levels of vitamin D in 49.9% of healthy Italian teenagers and correlated it to ethnicity, weight, and sun exposure [22]. A recent study in Brazil also analyzed laboratory results and found similar values of serum 25(OH)D (33.9% deficiency and 70.7% insufficiency) [23]. Other studies also detected a high prevalence of vitamin D deficiency in other populations around the globe, including countries with a sunny environment [24–26].

Our study also detected that vitamin D deficiency seems to be related to the age of the individuals. The geriatric population is highly prone to develop alterations in the mechanisms of 25(OH)D synthesis, due mainly to physiological changes related to the ageing process. The present study showed significant vitamin D deficiency in 70 and older individuals. The high prevalence of hypovitaminosis D in the elderly could be associated with changes in mineral bone density, decreased sun exposure, and secondary hyperparathyroidism [15]. The study by Quaggiotto et al. corroborated with the present study, showing from a laboratory databank analysis that high levels of 25(OH)D were detected in young individuals, while aged individuals presented the lowest values of 25(OH)D [20]. Other studies also suggest that vitamin D is prevalent among older people and could be a risk factor for the development of osteoporosis and osteomalacia [27–29]. Although many studies show a correlation between vitamin D deficiency and age, it is important to note that even healthy teenagers and children can bear vitamin D levels considered insufficient [24, 25].

The association between parathyroid hormone (PTH) and vitamin D may be an important determinant of bone remodeling, mainly in the elderly. A negative and significant correlation was found between PTH and 25(OH)D levels in the present study. Individuals with low vitamin D levels were those who had higher values of PTH, while individuals with high values of vitamin D showed low values of PTH. Similar results were observed in healthy individuals in Australia and Riga, and a value of 38 ng/mL was suggested as sufficient to avoid an increase in PTH [20, 30]. Other studies reinforce the negative correlation between vitamin D and PTH [31–33].

The Brazilian Society for Endocrinology and Metabolism, and other groups, suggests that vitamin D supplementation should not be recommended for the general population, but is indicated for individuals with high risk of developing hypovitaminosis and those with need to avoid sun exposure, as in skin cancer and systemic lupus erythematosus [13, 34]. Dietary supplement recommendations should be decided taking into consideration many factors, such as clinical condition, baseline nutrition, country development, and health status [35]. The two main choices for vitamin D supplementation are vitamin D2 (ergocalciferol) and vitamin D3 (cholecalciferol), and the therapeutic regimen of supplementation depends on several factors, such as the dosing regimen, severity of vitamin D deficiency, convenience for the dosing regimen, and safety [36, 37].

This study has some limitations. As the data was obtained from a laboratory databank, some variables were unavailable and have not been analyzed. As in laboratory forms only age and gender were recorded, we did not have information about the subject's medical history, including height, weight, and their possible clinical conditions. Other important information was also unable to be obtained, such as hours of sun exposure, use of sunscreen, and calcium and vitamin D dietary intake. These variables could at some extent contribute to serum vitamin D content. However, as large amount of data was analyzed, the results represent clear and valuable information.

## 5. Conclusions

The present study demonstrated a high prevalence of deficiency and insufficiency of vitamin D, mainly among older people but also in younger people, and a negative correlation between 25(OH)D and PTH levels was detected. This increase in vitamin D deficiency should not be underestimated, and 25(OH)D laboratory tests results should be used with other clinical and laboratory information to help achieve the best treatment decision for each patient. Other studies are needed to further define optimal reference levels of vitamin D for different individuals, healthy or unhealthy, and to lead to a better understanding of the complex mechanisms underlying hypovitaminosis and vitamin D importance in health and diseases.

## Figures and Tables

**Table 1:** Results for serum 25(OH)D levels observed by analysis of a clinical laboratory databank. Data are presented as mean ± standard deviation and median of 25(OH)D concentration in ng/mL.

Year	Mean ± SD	Median	P25	P75
2011	25.5 ± 11.0	24.6	19	30.6
2012	24.5 ± 8.9	23.9	19.1	29
2013	25.6 ± 9.1	24.8	20.1	30
*Total*	*25.3 ± 9.3*	*24.5*	*19.6*	*29.7*

P25 are Values for percentile 25 and P75 are values for percentile 75.

SD is Standard deviation.

**Table 2:** Percentage of serum 25(OH)D results according to reference values.

Reference value	*N*	%
<20 (deficiency)	28065	26.5%
20,1–30 (insufficiency)	52304	49.5%
30,1–100 (sufficiency)	25099	23.8%
>100 (increased)	120	0.11%
*Total*	*105588*	*100%*

**Table 3:** Parathyroid hormone concentrations according to vitamin D range. Values of PTH are presented as mean ± standard deviation.

Vitamin D (ng/mL)	PTH (pg/mL)^*∗*^
<20	31.2 ± 18.9
20–30	28.3 ± 13.7
30.1–100	25.5 ± 9.4
>100	23.4 ± 9.5

^*∗*^Significant correlation (*p* < 0.001).
